# Informiertes Vertrauen in Wissenschaft: Lehren aus der COVID-19 Pandemie für das Verständnis naturwissenschaftlicher Grundbildung (scientific literacy)

**DOI:** 10.1007/s42010-022-00159-6

**Published:** 2022-10-27

**Authors:** Rainer Bromme

**Affiliations:** Institut für Psychologie, Universität Münster, Fliednerstr. 21, 48149 Münster, Deutschland

**Keywords:** Wissen/Vorwissen, Soziale Medien, gesellschaftlich-wissenschaftliche Problemstellung, Gültigkeit, Überzeugung, Knowledge/prior knowledge, Social media, Socio-sceintific-issue, Validity, Belief

## Abstract

*Informiertes Vertrauen in Wissenschaft* ist nötig, damit die ‚Schnittstellen‘ für den Wissensfluss zwischen dem Alltagsverständnis der Bürger:innen über die Pandemie und dem sich dynamisch entwickelnden Wissensstand der Wissenschaften funktionieren. Das ist die Kernthese dieses Beitrags. Ohne Wissenschaft kann die COVID-19 Pandemie weder verstanden noch beherrscht werden und auch Bürger:innen müssen sich dafür mit Wissensangeboten aus der Wissenschaft auseinandersetzen. Bei einer solchen Problemlage sind diese Wissensangebote aber dynamisch, d. h. sie entwickeln sich weiter und sie sind eingebettet in normative Fragen. Außerdem konkurrieren sie mit pseudowissenschaftlichen Beiträgen. Als Nicht-Expert:innen müssen Laien deshalb entscheiden wem sie vertrauen können. In dem Beitrag wird das Konzept der *functional scientific literacy* als Voraussetzung von Urteilen des informierten Vertrauens beschrieben. In der Schule sollten die Wissensgrundlagen für Urteile des *informierten Vertrauens* vermittelt und eine rationale Beurteilung der Vertrauenswürdigkeit von wissenschaftsbezogenen Wissensangeboten eingeübt werden.

## Abstract (en)

*Informed trust in science* is necessary for the ‘interfaces’ within the flow of knowledge between citizens’ everyday understanding of the pandemic and the dynamically evolving state of knowledge in the sciences. This is the core thesis of this paper. Without science, the COVID-19 pandemic can neither be understood nor controlled, and for this to happen, citizens must engage with science based knowledge. However, such knowledge is dynamic (evolving and intertwined with normative issues). Furthermore, science based knowledge competes with pseudoscientific contributions. As non-experts, laypersons must therefore decide whom to trust. The paper describes the concept of *functional scientific literacy* as a prerequisite of informed trust. The knowledge bases for judgments of *informed* trust should be taught in school and judging rationally about the trustworthiness of science-related knowledge claims should be practiced.

## Einführung

Die COVID-19-Pandemie hat viele neue Herausforderungen für die Schule gebracht, noch mehr aber hat sie bereits vorher bestehende Bedarfe verstärkt und Defizite sichtbar gemacht. Dies gilt für die räumliche und digitale Ausstattung von Schulen. Es gilt ebenso für die organisatorische und pädagogische Gestaltung des Distanzlernens und hybrider Formate, die dafür sorgen, dass eine adäquate und bildungsgerechte Teilhabe an der Schule auch für solche Schüler:innen möglich ist, die nicht am Präsenzunterricht teilhaben können (Fickermann und Edelstein 2021; Huber et al. 2021). Solche Herausforderungen stehen zu Recht im Mittelpunkt der gegenwärtigen Diskussionen um allgemeinbildende Schulen im COVID-19-Kontext. Darüber hinaus wirft die COVID-19-Pandemie aber auch die Frage nach inhaltlichen und lernzielbezogenen Bedarfen auf, d. h. ob aus der Pandemie auch curriculare Folgerungen zu ziehen sind^1^. Etwas salopp gesagt: Der Ausbruch der Pandemie hat zuerst die Frage aufgeworfen, wie man sicherstellen kann, *dass* Lehrer und Schüler weiter miteinander sprechen (lehren und lernen) *können.* Die nächste Frage ist dann aber, *worüber* man denn angesichts der Pandemie sprechen (lehren und lernen) *sollte*.

Dieser Beitrag skizziert eine mögliche Antwort für den Themenbereich der wissenschaftlichen Grundbildung (*scientific literacy*) und dann noch spezieller, des Wissens über die Natur der Wissenschaft (*Nature of Science, NOS*). Daneben gibt es andere Themenbereiche, wie z. B. Sachwissen zum Alltags-Umgang mit der Pandemie, die auch Ansatzpunkte für eine Vermittlung wissenschaftlicher Grundbildung bieten, die hier aber nicht behandelt werden können.

## Die COVID-19-Pandemie hat den Bedarf nach einer *functional scientific literacy* für alle Bürger:innen deutlich gemacht

Ohne Wissenschaft kann die COVID-19-Pandemie weder verstanden noch beherrscht werden. Da es sich um eine komplexe Viruskrankheit mit einem breiten Symptomspektrum handelt, ist es noch nicht einmal möglich, sie ohne wissenschaftliche Methoden zu erkennen. Man kann auch nicht ohne Rückgriff auf die Wissenschaftssprache über die Pandemie sprechen. COVID-19, wie auch Synonyme, z. B. SARS-CoV‑2, sind Begriffe aus der Wissenschaftssprache wie auch viele sonstige Begriffe des gegenwärtigen öffentlichen Diskurses, z. B. *Inzidenzwert, Aerosole* oder *Impfdurchbrüche*. Politische Entscheidungen zum Umgang mit der Pandemie ebenso wie Verhaltensempfehlungen werden überwiegend durch Bezug auf Wissenschaft begründet. Surveys zu wissenschaftsbezogenen Einstellungen zeigen, dass die öffentlichen Erwartungen an die Leistungsfähigkeit der Wissenschaft zum Verständnis und zur Bekämpfung der Pandemie sehr hoch sind. Im Vergleich zu Messungen vor dem Ausbruch der Pandemie ist das allgemeine Vertrauen in Wissenschaft in Deutschland, aber auch in fast allen anderen Ländern, deutlich gestiegen (Algan et al. 2021; Bromme et al. 2022; WellcomeTrustGallup 2021). Tatsächlich ist die Pandemie eine globale Erfolgsgeschichte der Wissenschaft, zumindest in Bezug auf die unmittelbar gesundheitsbezogenen Fachgebiete und in Bezug auf ihre epistemische Funktion, also die *Produktion* gesicherten Wissens, (nicht aber, wenn es um die globale *Anwendung* dieses Wissens geht, wie die Defizite der globalen Versorgung mit Impfstoffen zeigen).

Die Pandemie hat der Öffentlichkeit einen Einblick in den Maschinenraum der Wissensproduktion gegeben, nicht nur – um in dem Bild zu bleiben – in die Auslieferungshalle für fertige Produkte. Es gibt eine große Bereitschaft vieler Wissenschaftler:innen ihre Ergebnisse zur Pandemie schnellstmöglich mit der Öffentlichkeit zu teilen. Die – eigentlich innerwissenschaftlich intendierte – Kommunikation ist öffentlich leicht zugänglich, da sie sich zunehmend sozialer Medien bedient. Die Orte der Lagerung von Zwischenergebnissen (z. B. pre-print Server) sind ebenfalls leicht zugänglich. Schließlich gibt es großes mediales Interesse an wissenschaftlicher Evidenz.

Zugleich wurden aber auch die Schwierigkeiten für Bürger:innen deutlich, sich in dem COVID-19 bezogenen Informationsangebot zu orientieren. Der Einblick in den Maschinenraum der Wissensproduktion bedeutete: Epidemiologie, Virologie wie auch die klinischen Fächer, die für die Behandlung von COVID-Erkrankungen zuständig sind, kommunizierten umfangreich über offene Fragen, Wissenslücken und Widersprüche. Auch nicht-medizinische Fächer, die sich mit den psychosozialen, ökonomischen und kulturellen Auswirkungen der Pandemie befassen, standen ebenfalls vor vielen neuen Fragen und konnten erst im Laufe der Zeit belastbare Daten liefern (z. B. zur Frage der psychosozialen Effekte auf Kinder). Die Zugänglichkeit dieser Diskurse hat der Öffentlichkeit auch die Komplexität der Problemstellungen deutlich gemacht. Die repräsentativen Erhebungen des Wissenschaftsbarometers fragen regelmäßig danach, ob Bürger:innen Wissenschaft und Forschung als zu komplex empfinden. Zu Beginn der Pandemie nahm der Anteil der Bürger:innen ab, die den Eindruck hatten, dass Wissenschaft und Forschung zu komplex für sie sei, um sie zu verstehen (gegenüber vergleichbaren Fragen in den Vorjahren). Dann, im Laufe der Pandemie verstärkte sich dieser Eindruck wieder (Bromme et al. 2022).

Durch das Internet und insbesondere die sozialen Medien, erhalten Bürger:innen eine Vielzahl von wissenschaftsbezogenen Informationen, die aber oft fragmentiert und auch vielfach keiner Qualitätskontrolle unterworfen sind (Höttecke und Allchin 2020). Das Internet ist auch der Raum für die Verbreitung von Falschinformationen (Ecker et al. 2022). So beteiligten sich nicht nur tatsächliche Fachleute an den öffentlichen Debatten über wissenschaftliche Evidenz zu COVID-19, sondern es gab auch vielfältige Fehlinformationen, die z. B. von sogenannten ‚Querdenkern‘ verbreitet wurden und die mit dem Anspruch, wissenschaftsbasiert zu sein, vorgetragen wurden. Zwar gab es auch schon vor der Entwicklung des Internets einen Chor an Stimmen zu den Fragen (z. B. Gesundheitsthemen), zu denen Bürger:innen wissenschaftsbasierte Informationen suchen. Aber durch das Internet ist dieser Chor dissonanter und umfangreicher geworden.

Die COVID-19-Pandemie ist also nicht nur eine (natur- und sozial-) wissenschaftliche Fragestellung, sondern auch eine gesellschaftlich-wissenschaftliche Problemstellung (Socio-Scientific-Issue, mehr dazu in Abschnitt 5). Viele praktische Maßnahmen erfordern normative Entscheidungen, z. B. darüber, welche Lasten von welcher Bevölkerungsgruppe zu tragen sind. Dabei sind die normativen und die wissenschaftlichen Fragen oft nur sehr schwer zu trennen. So ist z. B. die Frage nach der Rolle von Schulkindern für die Ausbreitung der Pandemie einerseits eine epidemiologische Frage, andererseits wurde sie in der öffentlichen Debatte auch immer im Zusammenhang mit der normativen Frage nach Schulschließungen diskutiert. Ein weiteres, subtileres Beispiel für die Vermischung von normativen und wissenschaftlichen Fragen betrifft die Indikatoren zur Modellierung des Infektionsgeschehens. So umfasst die Frage, ob man Maßnahmen zur Pandemiebekämpfung von Inzidenzraten, Klinikeinweisungen oder der Belastung von Intensivstationen abhängig machen sollte, einerseits die *wissenschaftliche* Problemstellung nach dem statistischen Zusammenhang zwischen diesen Parametern (der z. B. von dem dominanten Virustyp, aber auch von dem Impfstatus einer Population abhängt). Andererseits enthält die Frage nach den geeigneten Indikatoren auch einen *normativen* Aspekt, weil man z. B. bei der Überlastung der Krankenhäuser als Schwelle für restriktivere präventive Maßnahmen die Quote der dort versorgten Patienten als unabänderlich hinnimmt. Bildlich gesprochen: Solange keine Patienten auf den Fluren der Krankenhäuser untergebracht werden müssen, wird die Wahl der präventiven Maßnahmen nicht an dem Ziel orientiert, die Zahl der COVID-Erkrankungen noch weiter zu senken.

Zusammenfassend ist festzuhalten: Die COVID-19-Pandemie hat einerseits (Natur)-Wissenschaft im Alltag und im öffentlichen Diskurs sehr präsent gemacht hat. Anderseits hat die Dynamik der Wissensentwicklung und die Tatsache, dass es sich um Problemlagen handelt, bei denen politisch gesellschaftliche und wissenschaftliche Fragestellungen eng verknüpft sind, die Orientierung für Bürger:innen über wissenschaftlich begründete Gewissheiten sehr schwierig macht.

## Informiertes Vertrauen ist der Grundmodus der Begegnung von Bürger:innen mit Wissenschaft

Bürger:innen sind *epistemisch* abhängig von Expert:innen. Vertrauen ist deshalb nicht ein wünschenswerter Nebeneffekt (nice to have), wenn Bürger:innen sich mit Wissenschaft beschäftigen. Sie haben auf Grund der gesellschaftlichen Arbeitsteilung letztlich gar keine andere Möglichkeit als immer dann, wenn es um ‚wahres‘, belastbares wissenschaftliches Wissen geht, darüber als Vertrauensfrage in Bezug auf Expert:innen bzw. auf die Wissenschaft(en) zu urteilen. Deshalb ist *Vertrauen* (oder auch *fehlendes* Vertrauen) der Grundmodus der Begegnung von Bürger:innen mit Wissenschaft.

Das sei an einem Beispiel erläutert: Im Kontext von COVID-19 wollen Bürger:innen wissen, ob die neu entwickelten mRNA-Impfstoffe langfristige Wirkungen auf die Gene der geimpften Personen haben könnten. Es besteht ein Konsens in der Wissenschaft, dass dies nicht der Fall ist, aber es gibt sowohl Plausibilitätsüberlegungen (wie kann man so etwas ausschließen, wenn ein Impfstoff erst kürzlich entwickelt wurde?), wie auch vielfältige Stimmen im Internet, die eine solche schädigende Wirkung entweder direkt behaupten oder aber wenigstens für möglich halten.

Wie können Bürger:innen nun beurteilen, welche Aussagen zu den Nebenwirkungen von mRNA-Impfstoffen wahr sind? Sie haben zwei Möglichkeiten:

- ***Direkte Gültigkeitsbeurteilung*:** **Was ist wahr? **(first-hand evaluation) Das erfordert das Evaluieren der Plausibilität (subjektive Wahrheit) der Aussage, z. B. anhand von eigenen Empfindungen, Beobachtungen, der Prüfung der logischen Konsistenz und der Kohärenz mit eigenem Vorwissen und sonstigen verfügbaren Informationen.
- ***Indirekte Gültigkeitsbeurteilung: *Wem kann man glauben? **(second-hand evaluation) Evaluieren der Vertrauenswürdigkeit von Experten, welche die Gültigkeit der Aussage behaupten/begründen.

Diese beiden Möglichkeiten erfordern eine metakognitive Entscheidung darüber, welche Informationen und Vorannahmen beachtet und verarbeitet werden: über die Sache, um die es geht oder über die Quelle der Aussage. Den größeren Teil unseres Wissens und unserer Überzeugungen über uns selbst und unsere natürliche und soziale Umwelt erwerben wir nicht durch persönliche unmittelbare Erfahrungen, sondern durch ‚Hörensagen‘ von anderen. Bereits kleine Kinder (ab 2–3 Jahre) haben die Fähigkeit, die Vertrauenswürdigkeit von Informationsquellen zu beurteilen (Harris 2012). Dennoch sind wir epistemische Individualisten (Levy 2019). Es gibt eine intuitive Präferenz für direkte Plausibilitätsurteile (Abgleich mit dem eigenen Vorwissen), d. h. wir prüfen die Wahrheit von Aussagen danach, ob sie zu dem passen, was wir selbst erleben (sehen, hören, schmecken, fühlen) oder für plausibel halten, weil es zu unserem Vorwissen passt. Nur bei Inkonsistenzen zwischen Vorwissen und zu beurteilender Aussage wird auf Merkmale der Quelle der Aussage geachtet (Bromme et al. 2018). Die intuitive Präferenz für die erste Möglichkeit ist im Alltag notwendig und effektiv. Wenn es jedoch um Aussagen geht, deren Gültigkeit nur mit viel Fachkenntnis zu überprüfen ist, ist diese intuitive Präferenz problematisch.

Das Beispiel der Frage nach den mRNA-Impfstoffen zeigt, dass Bürger:innen (sofern sie nicht selbst eine Fachausbildung haben, die impfbezogene Themen umfasst) nicht alleine auf eine direkte Gültigkeitsbeurteilung in der Sache setzen können. Sie können jedoch nur auf indirekte Gültigkeitsprüfungen (Vertrauensurteile) setzen („Ich verstehe nicht was mRNA-Impfstoffe sind, vertraue aber dem, was das RKI zur Frage der Langzeitwirkungen sagt“).

Oder sie können beide Strategien verfolgen („Ich suche die Quellen, denen ich vertraue, lese auf den Websites, die mir glaubwürdig erscheinen und höre Podcasts mit Expert:innen, denen ich vertraue, (z. B. Corona Update des NDR oder www. Infektiopod), und lasse mir dort erklären, ob und warum die mRNA-Impfstoffe so sicher sind, dass keine langfristigen Effekte für meine Gene zu erwarten sind“).

In diesem Fall erfolgt die Gültigkeitsbeurteilung im wechselseitigen Abgleich von sach- und quellenbezogenen neuen Informationen und bereits bestehendem Wissen (Corner und Hahn 2009). Wenn Bürger:innen den zweitgenannten Weg gehen, entsteht subjektiv möglicherweise dennoch der Eindruck, dass sie nur auf Grund des eigenen Verständnisses urteilen, das sie sich durch die Auseinandersetzung mit dem Wissen vertrauenswürdiger Expert:innen erworben haben. Dieser Eindruck ist umso stärker, je einfacher und damit verständlicher diese Erklärungen der Expert:innen waren. Das kann zu der Illusion einer Überwindung der epistemischen Abhängigkeit führen (easiness effect; Scharrer et al. 2016, 2014). Tatsächlich liefert das – bildlich gesprochen – *lokale* Verständnis des Gebiets rund um das Thema mRNA noch kein Verständnis der *ganzen *Landschaft der Immunologie und anderer Fachgebiete, auf der die Gültigkeit der *lokalen *Aussagen, die man nun verstanden hat, beruht. Die Bürger:innen bleiben also epistemisch abhängig davon, dass sie auf die Expert:innen vertrauen müssen, die um den Begründungskontext dessen wissen, was sie nun selbst auf Plausibilität (mit der ‚Was ist wahr?‘ Strategie) überprüfen können^2^.

In diesem Sinne ist hier Vertrauen (oder auch fehlendes Vertrauen) als Grundmodus der Begegnung von Bürger:innen und Wissenschaft gemeint. Vertrauensurteile ermöglichen also den Umgang mit Grenzen des Verstehens und Urteilens, das auf der Basis der eigenen Anschauung und/oder der eigenen Vorkenntnisse^3^ geschieht.

Die Pandemie hat aber auch viele Beispiele dafür geliefert, dass man *der Wissenschaft* nicht blind vertrauen kann. Zum einen gibt es nicht ‚die‘ Wissenschaft, sondern es gibt insbesondere zu aktuellen Fragen sich verändernde Aussagen und es gibt natürlich auch viele Stimmen, die nur behaupten, ihre Aussagen seien nach den Regeln guter wissenschaftlicher Praxis begründbar. Oben wurde auch bereits erläutert, dass sich bei Socio-Scientific-Issues häufig wissenschaftliche und normativ-politische Fragen vermischen. Auch deshalb ist ein kritisches Urteil darüber notwendig, wem man vertrauen kann, weil sie/er diese beiden Arten von Fragen zu trennen vermag.

Es kann also nur um ein kritisches Vertrauen gehen, das wir hier als *informiertes Vertrauen* (Bromme 2020a) bezeichnen, um hervorzuheben, dass die Vertrauensurteile wissens- und verstehensbasiert erfolgen sollten. Das dafür notwendige Wissen und Verstehen sollte in der allgemeinbildenden Schule vermittelt werden, weil auf diese Weise möglichst allen Bürger:innen ein rationaler Umgang konkurrierenden Geltungsbehauptungen ermöglicht wird deren Gültigkeit sie – aus eigener Sachkenntnis heraus – nicht abschließend beurteilen können. Es bedarf also eines Grundbestandes an Wissen über die Kriterien von Vertrauenswürdigkeit von Wissenschaft (als System und als Personen). Und es bedarf der Einübung in der Anwendung dieser Kriterien, damit diese Vertrauensurteile rational erfolgen können (Keren 2018; Blancke und Boudry 2022). Diesen Grundbestand an Wissen über die Kriterien der Vertrauenswürdigkeit von Wissenschaft betrachten wir als wichtigen Teil einer *functional scientific literacy*. Die Einübung derartiger wissensbasierter Vertrauensurteile ist in diesem Sinn eine Kernaufgabe des (natur)^4^-wissenschaftlichen Unterrichts. In den nächsten beiden Abschnitten wird skizziert was thematisch zu diesem Grundbestand an Urteilsfähigkeit und Wissen gehört.

## Informiertes Vertrauen erfordert abwägendes Urteilen und Wissen über Wissenschaft

### Abwägendes Urteilen

Wie beurteilen wir im Alltag, ob eine Sachaussage wahr oder falsch ist? Sozial- und kognitionspsychologische Forschung zeigt, dass dieses Urteil auf Schlussfolgerungen aus den jeweils verfügbaren Informationen basiert, die bestimmten Heuristiken bei der Auswahl und Gewichtung dieser Informationen folgen. Mit *abwägendem* Urteilen bezeichnen wir Urteilsprozesse, in denen auch mögliche Gegenargumente oder Fehlermöglichkeiten bei der Nutzung dieser Heuristiken berücksichtigt werden. Das ist nicht immer der Fall; diese Heuristiken werden in der psychologischen Literatur deshalb vor allem im Kontext fehlerhafter Schlussfolgerungen erforscht und diskutiert, aber es ist wichtig festzuhalten, dass ihre Anwendung in vielen Situationen rational ist und zu sachlich korrekten Urteilen führt (Ecker et al. 2022). Drei Beispiele für diese Heuristiken (Brashier und Marsh 2020): Weil wir – insgesamt, d. h. im Laufe unseres Lebens – mehr wahre als falsche Aussagen hören, ist es rational davon auszugehen, dass eine neue Sachaussage eher wahr als falsch ist (*base-rate heuristic*). Wir folgen unserem Gefühl, z. B. der erlebten Einfachheit der Verarbeitung der Informationen bei Sachaussagen (*fluency effect*, eine Variante der *emotion heuristic*), d. h. die Heuristik lautet, dass eine Sachaussage eher wahr ist, wenn wir sie leichter verarbeiten können, z. B. erscheint uns ein Satz plausibler, wenn er gut lesbar gedruckt ist als wenn er schwer zu entziffern ist. In unserem Zusammenhang der Beurteilung der Gültigkeit von wissenschaftsbezogenen Geltungsbehauptungen ist die wichtigste Heuristik: Wir halten eher das für wahr, was mit dem übereinstimmt, was wir bereits wissen (als wahr erlernt/beurteilt haben; *content-memory heuristic*). Wie oben bereits erwähnt, orientieren wir uns an dem, was wir über die Glaubwürdigkeit und Zuverlässigkeit der Quelle der zu beurteilenden Sachaussage wissen (*source-memory heuristic*) erst dann, wenn wir Anlass zu der Vermutung haben, dass eine Sachbehauptung nicht zutrifft (weil sie z. B. im Widerspruch zu dem steht, was wir bereits für wahr halten).

Die Heuristiken können gerade im Zusammenwirken zu Fehlurteilen führen. Ein Beispiel haben wir oben erwähnt, den *easiness effect* bei der Gültigkeitsbeurteilung schwieriger wissenschaftsbasierter Sachaussagen, deren Gültigkeit man als Laie eigentlich nicht beurteilen kann. Wenn die Sachaussagen verständlich erscheinen, sinkt die Wahrscheinlichkeit, dass man sich noch weiteren Expertenrat einholt, d. h. man verlässt sich eher auf das Erleben des Verstehens und die Anknüpfung an das eigene Vorwissen; das ist also ein Beispiel für das Zusammenwirken der *emotion heuristic* und der *content-memory heuristic*. Wenn bereits Überzeugungen zu dem jeweiligen Sachverhalt bestehen (z. B. zur Frage der Ursache des Klimawandels), dann tritt dieser Effekt aber nur auf, wenn die leichter verständliche Sachaussage in Übereinstimmung mit diesen Vorüberzeugungen steht (Scharrer et al. 2021).

Der *easiness effect* lässt sich durch einen expliziten Hinweis, dass es sich in der Sache um eine komplexe Fragestellung handelt, abmildern (Scharrer et al. 2014). Dieser Befund steht in Übereinstimmung mit anderen Studien, die gezeigt haben, dass sich die fehlerhafte Anwendung dieser Heuristiken durch explizite Hinweise reduzieren lässt. So befördert z. B. die Aufforderung ‚to think like fact checkers‘ die Bereitschaft, eine falsche Sachbehauptung, die man gelesen hat, bezüglich ihres Wahrheitsgehalts zu überdenken (Brashier et al. 2020). Ebenso kann die Aufforderung, die Beurteilung einer eben gelesenen Zeitungsschlagzeile bezüglich ihres Wahrheitsgehalts noch einmal zu überdenken, dazu beitragen, dass die Aussagen kritischer und damit auch realistischer beurteilt werden (Bago et al. 2020). Die Autoren finden diese Wirkung allerdings nur sehr eingeschränkt, wenn es um Verschwörungstheorien geht (Bago et al. 2022). Die Wirkungen solcher Aufforderungen zu abwägendem und damit auch langsameren Urteilen werden im Kontext von Zwei-Prozess-Modellen der Informationsverarbeitung interpretiert, in denen zwischen einer schnellen, oberflächlichen und einer langsameren, tieferen Verarbeitung unterschieden wird. Allerdings schützt ein langsameres und überlegteres Vorgehen nicht automatisch vor der Übernahme von falschen Informationen. Wenn es darum geht das eigene Weltbild gegen abweichende Informationen zu schützen, kann diese Form der überlegteren Informationsverarbeitung auch zur Abwehr korrekter Informationen beitragen; siehe dazu die erwähnten Übersichtsdarstellungen von Brashier und Marsh (2020) und Ecker et al. (2022). Es kommt also auch auf den Inhalt dessen an, was bei der langsameren und sorgfältigen Abwägung reflektiert wird. Dafür sprechen auch Befunde zu dem sogenannten ‚Impfen‘ gegen Falschinformationen (*inoculation theory*; Lewandowsky und van der Linden 2021). Dabei werden entweder bereits vor der Begegnung mit einer Falschinformation (pre-emptive measure) oder im Anschluss daran Warnungen vor Fehlinformationen und Aufklärung über die Quellen solcher Fehlinformationen gegeben. Eine solche Vorwarnung umfasst zwei Elemente; zum Beispiel im Kontext des Klimawandels: Den Hinweis darauf, dass man bei diesem Thema vielfach mit gezielten Falschinformationen rechnen muss. Und eine Erläuterung von Techniken der Falschinformation, wie z. B. die verzerrende Auswahl von solchen Forschungsergebnissen, die zu der eigenen Position passen (cherry-picking; Ecker et al. 2022). Diese Autoren weisen übrigens zu Recht darauf hin, dass solcheMaßnahmen des ‚Impfens‘ gegen Falschinformationen eine enge Verwandtschaft mit der Idee der Förderung des ‚kritischen Denkens‘ haben^5^.

Die Forschung zur *inoculation theory* bietet gute empirische Belege dafür, dass sowohl sehr inhaltsspezifische wie auch etwas allgemeinere Warnungen und Informationen über die Akteure, wie auch die Strategien der Verbreitung von Fehlinformationen, dazu beitragen können, dass Fehlinformationen weniger geglaubt werden und auch weniger stark im Gedächtnis präsent sind, wenn über den jeweiligen kritischen Sachverhalt weitere Informationen verarbeitet werden.

In der hier skizzierten Forschung wird üblicherweise das Urteil über die Gültigkeit von Sachaussagen (Faktenwissen) untersucht, jedoch sind diese Befunde auch aufschlussreich für das Urteil über die Vertrauenswürdigkeit von Quellen, weil es eine Wechselbeziehung zwischen der zugeschriebenen Glaubwürdigkeit einer Aussage und der Vertrauenswürdigkeit der Quelle dieser Aussage gibt und weil die Heuristiken auch direkt bei der Quellenbewertung wirksam sind. Zusammenfassend können wir damit festhalten, dass für informierte Vertrauensurteile ein abwägendes, eher langsames Urteilen eine gute Voraussetzung bietet, weil es die Wahrscheinlichkeit erhöht, Urteilsfehler zu vermeiden. Aber der langsamere und sorgfältigere Prozess benötigt auch, bildlich gesprochen, das richtige Material, d. h. es kommt auch auf das Wissen darüber an, warum und in welcher Hinsicht Wissenschaft und Wissenschaftler:innen vertrauenswürdig sind. In der Begrifflichkeit der eben referierten Forschung formuliert: Man benötigt Wissen darüber, welche Informationen (cues) über Wissenschaftler:innen/wissenschaftliche Institutionen für ihre Vertrauenswürdigkeit sprechen.

### Wissen über Wissenschaft

Was also ist für *informiertes* Vertrauen wichtig zu wissen, d. h. worüber sollten Schüler:innen etwas lernen, um auf Urteile des *informierten* Vertrauens vorbereitet zu sein? Um diese Frage zu beantworten, schlagen wir vor, sich an den Dimensionen von Vertrauen zu orientieren, die in dem Vertrauensmodell von (Mayer et al. 1995) unterschieden werden, das sind *expertise, benevolence* und *integrity; *mehr dazu, ebenfalls am Beispiel von COVID-19 in Bromme und Hendriks (2022). Diese Merkmale konnten als Dimensionen von Vertrauenszuschreibungen zu Wissenschaftler:innen auch empirisch unterschieden werden (Hendriks et al. 2015). *Expertise*: Wissenschaftler:innen haben Fähigkeiten und Erfahrungen zur Erkenntnisgewinnung und Problemlösung; *Integrität*: Wissenschaftler:innen halten sich an begründete Regeln der Wahrheitssuche; *Benevolenz*: Wissenschaftler:innen haben den Nutzen für andere (die Öffentlichkeit) bei ihrer Arbeit im Blick.

An diesen Dimensionen kann man sich auch orientieren, wenn es um Vertrauen in Institutionen oder, noch allgemeiner, die Wissenschaft als System geht und nicht nur um einzelne Wissenschaftler:innen. So ist es für Urteile über die *Expertise*, man könnte auch sagen, über die Erkenntnis und Leistungsmöglichkeiten der Wissenschaft, wichtig, ein Verständnis von Wissenschaft als Prozess zu vermitteln, der durch Institutionen und Personen in Gang gesetzt wird. Dafür wiederum wichtig ist ein Grundverständnis über Fächerstrukturen (wer ist wofür zuständig?) und über Institutionen der Wissenschaft (wer produziert das Wissen?). Im Kontext der COVID-Pandemie wurde das sehr deutlich. Die Fächerdifferenzierungen innerhalb der Medizin (z. B. zwischen Epidemiologie, Virologie, Pneumologie) bekamen plötzlich eine öffentliche Bedeutung für die Beurteilung der Frage, ob Wissenschaftler:innen für die Themen, zu denen sie sich äußern, auch tatsächlich kompetent sind. Die Wissensgrundlagen zur Beurteilung der *Integrität* im eben beschriebenen Sinne betreffen die methodischen Regeln und die sozialen Praktiken der Wahrheitssuche selbst. Hier ist z. B. Wissen über kooperative Prozeduren zur Wahrheitsfindung (z. B. peer review) hilfreich. Im Kontext der COVID-Pandemie wurde z. B. die Unterscheidung zwischen pre-print Publikationen und begutachteten Publikationen auch für die Öffentlichkeit relevant, weil – durchaus aus guten Gründen – plötzlich sehr viele Arbeiten auf pre-print Servern gestellt und dort auch für die Öffentlichkeit zugänglich wurden (Fraser et al. 2021). Für ein informiertes Urteil über das Vertrauen in Wissenschaft ist es hilfreich diese Unterscheidung zu kennen. Dies ist ein Beispiel für Wissen über Wissenschaft, das sich auf die konkrete Wissenschaftspraxis in einem bestimmten Kontext (hier: die Pandemie) bezieht. Dabei reicht bereits eine kurzer erklärender Hinweis, um eine kritische Beurteilung durch Laien anzuregen (Wingen et al. 2022). Ein anderes Beispiel ist Wissen über die Finanzierung von Forschung, das einen großen Effekt auf das Vertrauen in Wissenschaft hat. Wissenschaft, die an öffentlich finanzierten Institutionen durchgeführt wird, genießt mehr Vertrauen als private (z. B. industriell finanzierte) Forschung. Dies ist ein stabiler, in vielen Surveys (z. B. dem deutschen, schweizerischen und schwedischen Wissenschaftsbarometer) bestätigter Befund, der für die Dimension *Benevolenz* wichtig ist. Auch hierbei ist Wissen wichtig, denn diese Unterscheidung ist ohne Vorkenntnisse über die konkreten Verhältnisse der Forschungsfinanzierung nicht so einfach zu treffen. Wenn man z. B. kein Wissen darüber hat, dass Forschungsfinanzierung an Universitäten in großem Umfang zwar extern, aber indirekt dennoch durch öffentliche Mittel geschieht (z. B. durch die DFG), erscheint die große Abhängigkeit von Drittmitteln möglicherweise problematischer als sie es ist.

## Die COVID-19-Pandemie als Beispiel für die Vermittlung von* functional scientific literacy*- und zugleich als Anlass dieses Konzept neu zu denken

Wissenschaftsverständnis ist nötig für die Teilhabe von Bürger:innen in einer Wissensgesellschaft, weil Bürger:innen in ihrem privaten wie auch in ihrem gesellschaftlichen Leben in vielfältiger Weise auf wissenschaftliches Wissen angewiesen sind (Bromme und Goldman 2014). Es ist also nahliegend, die COVID-19-Pandemie als Anschauungsbeispiel für die Vermittlung von grundlegendem Wissenschaftsverständnis zu nutzen. Problemstellungen, die sowohl eine wissenschaftliche wie auch eine gesellschaftliche Dimension enthalten, werden in der fachdidaktischen Literatur der MINT Fächer als *Socio-Scientific-Issues* (SSI) bezeichnet. SSI gelten insbesondere als gut geeignet, um die Struktur und Arbeitsweise von (Natur‑) Wissenschaft zu vermitteln, was als *Nature of Science* Verständnis (NOS) beschrieben wird (Neumann 2022; Sadler et al. 2004). Bereits 2009, damals unter Bezug auf die erste SARS-CoV-Epidemie, die 2002–2003 auftrat, haben Wong et al. (2009) vorgeschlagen, SARS als Kontext für die Vermittlung eines NOS Verständnisses zu nutzen: ‚*Turning crisis into opportunity*‘; in analoger Weise leiten Maia et al. (2021) Themen aus der COVID-19 Pandemie für die Vermittlung von NOS Kenntnissen ab.

Der Umgang von Bürger:innen mit derartigen Socio-Scientific-Issues erfordert ein grundlegendes Verständnis von (Natur‑) Wissenschaft (scientific literacy), das es ihnen erlaubt sich in den vielfältigen Informations- und Deutungsangeboten mit Wissenschaftsbezug zu orientieren. Anknüpfend an (Tabak 2015, 2018), siehe auch (Kienhues et al. 2018), bezeichnen wir dieses Verständnis als *functional scientific literacy.* Die Entwicklung des Konzepts der *functional scientific literacy* wurde durch die – oben im COVID-19 Kontext beschriebene – Bedeutung des Internets für die Begegnung von Bürger:innen mit Wissenschaft befördert. Der Kern dieses Konzeptes von wissenschaftlicher Grundbildung ist die Orientierungsfunktion für den Umgang mit wissenschaftlichem Wissen für Bürger:innen.

Die COVID-19-Pandemie hat den Bedarf nach einer derartigen *functional scientific literacy* möglichst für alle Bürger:innen deutlich gemacht und sie ist auch ein Anlass, dieses Konzept weiterzuentwickeln. Nachfolgend wird begründet, dass es dabei im Kern um die Bildungsvoraussetzungen für* informiertes Vertrauen in Wissenschaft *gehen sollte.

Die oben erwähnten fachdidaktischen NOS Ansätze haben einen Schwerpunkt auf den – im weitesten Sinne – methodischen Grundlagen der Wahrheitsfindung. Sie beschreiben viel genauer als es hier möglich ist, das Wissen, das zur Beurteilung von *Integrität* notwendig ist. Der hier vorgelegte Vorschlag *functional scientific literacy* auf Wissen und Verständnis für Urteile des *informierten Vertrauens* auszurichten, enthält nun aber eine gewisse Akzentverschiebung von *Nature of Science* zu (um im Englischen zu bleiben), *Facts and Findings about Science in Society*. Das ist hier mit ‚gewisser Akzentverschiebung‘ bewusst unscharf formuliert, weil es in der fachdidaktischen Literatur unterschiedliche Akzente im Verständnis von *Nature of Science* gibt, nämlich solche, die sich vor allem an einem wissenschaftsphilosophisch geprägten Verständnis der *Natur der Wissenschaft* orientieren und solchen, die ergänzend die Praxis von *Wissenschaft in der Gesellschaft* im Blick haben, so wie es auch hier vorgeschlagen wird (z. B. Maia et al. 2021; Neumann 2022). Auch Höttecke und Allchin (2020) bieten ein Beispiel für eine solche Erweiterung des NOS Konzepts, indem sie die mediale Vermittlung von Wissenschaft in den Blick nehmen. Auch sie gehört zu *Nature of Science*, nun aber verstanden als *Science in Society*.

Warum ist es nun, wie hier vorgetragen wird, so wichtig *functional scientific literacy* auf die Fähigkeiten zu Urteilen des *informierten Vertrauens* auszurichten? Warum nicht nur auf die Verbesserung des Sachverständnisses über die Pandemie und über die Wissenschaften zu ihrer Erforschung? Zeigt nicht die Pandemie (zumindest in Deutschland), dass es möglich ist, in kurzer Zeit virologisches, epidemiologisches und immunologisches Basiswissen so zu verbreiten, dass auch Nichtfachleute recht selbstverständlich mit Fachbegriffen aus diesen Gebieten kommunizieren^6^ können? Das große Interesse an dem, fachlich sehr anspruchsvollen, NDR Podcast Corona Update^7^ (mit den Profs. Ciesek und Drosten) deutet auf diese Möglichkeit hin.

Die Antwort auf diesen Einwand wurde oben bereits skizziert: Letztlich, d. h. wenn es um besonders umstrittene oder um besonders neue und damit auch wieder veränderliche Wissenschaft geht, können neue wissenschaftliche Ergebnisse mit Hilfe dieses neu gewonnenen pandemiebezogenen Wissens möglicherweise von vielen Bürger:innen in Umrissen verstanden werden. Sie können aber deshalb noch nicht von den Bürger:innen auch gegen andere, alternative Behauptungen verteidigt werden.

Dies soll nachfolgend am Beispiel des Umgangs mit dem sogenannten ‚post truth‘ Phänomen (Kienhues et al. 2020) erläutert werden. Es gibt eine Variante der Wissenschaftsleugnung, die die subjektive Erfahrung und das subjektive Erleben als letzte Instanz für die Frage der Wahrheit wissenschaftlicher Aussagen postuliert (Bromme 2020b). Wenn sogenannte ‚Querdenker‘ darauf bestehen, dass es für die empirische Frage der Häufigkeit von Nebenwirkungen eines Impfstoffes eine persönliche Wahrheit^8^ gibt, so ist das eine Form der Wissenschaftsleugnung. Es ist auch dann Wissenschaftsleugnung, wenn sie den Gesprächspartnern, die wissenschaftsbasierte Auffassungen vertreten, zubilligen, eben an deren eigene, subjektive Wahrheit zu glauben. Im Ergebnis führt eine solche Perspektive auf Fragen, die man wissenschaftlich beantworten kann, zu einer Fragmentierung des gemeinsamen Grundbestandes von Weltwissen, also ‚der kleinsten gemeinsamen Wirklichkeit‘ (Nguyen-Kim 2021). Soziologen und Politologen bezeichnen das als epistemische Tribalisierung (Bogner 2021; Zürn 2022).

Der Umgang mit dieser Art von Argumenten nur auf der Ebene der jeweiligen Sachfrage kann für Bürger:innen (Laien) recht schwierig werden, wenn sich die Grenzen der eigenen Sachkompetenz herausstellen^9^. Dann wird es wichtig* rational* erklären zu können, und noch wichtiger, für sich selbst zu wissen, warum man *der *Wissenschaft bzw. einzelnen Wissenschaftler:innen vertraut und ihnen deshalb auch bezüglich der Sachaussage (mRNA-Impfstoffe haben keine Effekte auf die Gene des Geimpften) *glaubt*. Das Wissen um die Grenzen des Alltagsverständnisses und um den Unterschied von Alltagsverständnis und wissenschaftlichem Verständnis gehört zur Anerkennung der *epistemischen Autorität* von Wissenschaft. *Functional scientific literacy* mit dem Schwerpunkt auf informiertem und das heißt auch kritischem Vertrauen in Wissenschaft macht es möglich, um die Grenzen des eigenen Verstehens wissenschaftlicher Erkenntnisse zu wissen, und dennoch einen rationalen Zugang zu einer von der Wissenschaft beschriebenen Welt zu haben.

## Footnotes

1 In analoger Weise fragt z. B. (Terhart 2022) nach den curricularen Folgerungen, die sich aus dem Phänomen ‚post-truth‘ ergeben. Die curricularen Folgerungen aus der COVID-19 Pandemie kann man auch im Zusammenhang mit der Frage nach dem Umgang mit ‚post-truth‘ Phänomenen sehen.

2 Mit dieser Feststellung ist nicht ausgeschlossen, dass Bürger:innen sich auch umfassende Expertise selbst aneignen, das ist bei entsprechendem zeitlichen Aufwand, guten Bildungsvoraussetzungen (zum Beispiel ist die meiste einschlägige Fachliteratur auf Englisch) und Zugang zur Fachliteratur durchaus möglich. Dann aber erwerben sie die Fachexpertise, von der Bürger:innen im Regelfall epistemisch abhängig sind; sie werden selbst zu Expert:innen und deshalb widerlegen solche Beispiele auch nicht das voranstehende Argument.

3 Auch innerhalb der Wissenschaft, also nicht nur zwischen Expert:innen und Laien, ist Vertrauen notwendig, weil es auch innerhalb der Wissenschaft eine Arbeitsteilung mit epistemischer Abhängigkeit gibt (Wilholt 2013).

4 Weil das am besten fächerübergreifend erfolgt (man kann und sollte über Wissenschaft auch in den sozialwissenschaftlichen Fächern sprechen) wird der Bezug zu den Naturwissenschaften hier in eine Klammer gesetzt.

5 Es gibt inzwischen diverse Ansätze für das Einüben einer kritischen Beurteilung von Quellen, die man bei der Internetsuche findet, z. B. Bråten et al. (2019), Stadtler et al. (2016).

6 Noch steht aber – nach meiner Kenntnis – eine empirische Untersuchung der Reichweite dieser Transformation von pandemiebezogenem Spezialwissen in den Bereich des Allgemeinwissens noch aus.

7 Bis zum 22.02.2022 gab es fast 135 Mio. Zugriffe seit dem Start des Podcasts (Anfang 2020), verteilt auf 110 reguläre Folgen und 15 Sonderfolgen. Während des ersten Lockdowns in der Pandemie gab es zeitweilig mehr als 2000 Emails von Hörer:innen pro Tag. (Persönliche Auskunft von K. Hennig, Wissenschaftsredaktion NDR Info).

8 Bogner (2021) vermutet, dass in diesem Fall die Impfgegner eigentlich nur auf ihrer subjektiven Bewertung der Impfoption bestehen. Diese ist selbstverständlich eine legitime persönliche Entscheidung, keine Frage der wissenschaftlichen Wahrheit. Selbst wenn diese Vermutung empirisch zutrifft, bleibt es notwendig, auf der Möglichkeit einer subjektunabhängigen ‚Wahrheit‘ in dieser Frage zu bestehen. Für viele Menschen ist für diese subjektive Entscheidung über die Impfoption auch die Faktenfrage nach dem tatsächlichen Risiko relevant. Um darüber überhaupt sprechen und sich rational verständigen zu können, ist es notwendig, diese Frage von der subjektiven Impfoption zu unterscheiden.

9 Es ist auch kein Ausweg, bei der epistemischen Tribalisierung sozusagen mitzumachen und nur darauf zu beharren, dass man selbst eben zu der Gruppe gehört, die an die Wissenschaft glaubt.
